# Morbidity and Quality of Life in Bladder Cancer Patients following Cystectomy and Urinary Diversion: A Single-Institution Comparison of Ileal Conduit versus Orthotopic Neobladder

**DOI:** 10.5402/2012/342796

**Published:** 2012-02-06

**Authors:** Barbara Erber, Mark Schrader, Kurt Miller, Martin Schostak, Daniel Baumunk, Anja Lingnau, Andres Jan Schrader, Florian Jentzmik

**Affiliations:** ^1^Department of Urology, Charité-University Medicine Berlin, 10117 Berlin, Germany; ^2^Department of Urology, University Hospital Ulm, Prittwitzstrasse 43, 89075 Ulm, Germany; ^3^Department of Urology, University Hospital Magdeburg, 39120 Magdeburg, Germany

## Abstract

*Objective*. To evaluate and compare noncontinent and continent urinary diversion after radical cystectomy in patients with bladder cancer. *Methods*. A total of 301 patients submitted to radical cystectomy at the Charité-University Hospital Berlin from 1993 to 2007 including 146 with an ileal conduit and 115 with an ileal neobladder. Clinical and pathological data as well as oncological outcome were retrospectively analyzed and compared. Quality of life was analyzed using the EORTC QLQ-C30 and BLM30 questionnaires. *Results*. 69.1% and 69.6% of all patients who received an ileal conduit and ileal neobladder, respectively, developed early complications. The two groups differed significantly concerning the occurrence of postoperative ileus (*P* = 0.02) favoring patients who received an ileal conduit but not with regard to any other early-onset complication evaluated. Patients with ileal neobladder had a significantly better global health status and quality of life (*P* = 0.02), better physical functioning (*P* = 0.02), but also a higher rate of diarrhoea (*P* = 0.004). *Conclusion*. Cystectomy with any type of diversion remains a complication-prone surgery. Even if the patient groups are not homogeneous in all respects, there are many arguments in favor of the ileal neobladder as the urinary diversion of choice.

## 1. Introduction

Radical cystectomy with bilateral lymphadenectomy and subsequent urinary diversion is regarded as the gold standard treatment for muscle-invasive bladder cancer without detectable hematogenous or lymphogenous metastases [1, 2]. The intervention should be preceded by detailed patient counseling on the advantages and disadvantages of different types of urinary diversion. While carefully considering oncological safety as well as early and late complications, physicians aim to individually determine what type of urinary diversion interferes least with the patient's personal lifestyle in order to achieve the best possible quality of life and overall treatment outcome in each particular case [3]. The advantages of continent urinary diversion are evident [4–6], though some studies have expressed the opinion that noncontinent urinary diversions are superior concerning potential advantages such as a faster and easier surgical technique, fewer complications, a lower reoperation rate, and thus a reduced morbidity [7, 8]. Taking these objections into account, the aim of this investigation was to critically evaluate the advantages and disadvantages of both procedures in order to have a contemporary basis for patient counseling.

## 2. Patients and Methods

### 2.1. Patient Population

From January 1993 to August 2007, 301 patients with bladder cancer underwent radical cystectomy with subsequent urinary diversion in the Department of Urology at Charité Campus Benjamin Franklin. The patient population comprised 225 men (75%) and 76 women (25%). The types of urinary diversion were as follows: ileal conduit in 146 cases (49%), ileal neobladder in 115 (38%), Mainz pouch I in 25 (8%), ureterocutaneostomy 10 (3%), and other diversions in 5 cases (1.7%). To enable statistical assessment of patient populations comparable in size, the study focuses on the analysis of ileal conduit (group 1) and ileal neobladder (group 2) urinary diversions. Data were retrospectively collected from the clinical records. A written inquiry was conducted on the quality of life. The inquiry was addressed to all patients who underwent radical cystectomy for primary bladder cancer from 1993 to 2007 and for whom there were no death data. Patients whose further clinical course was incompletely documented received a written inquiry regarding relapse. Comorbidity was assessed using the Charlson Comorbidity Index [9], which is weighted and takes into account the number and severity of comorbidities. Patient and tumor characteristics are summarized in Table 1. All early-onset complications were recorded, categorized, and then further grouped into categories as outlined in Table 2.

### 2.2. Radical Cystectomy and Urinary Diversion

Radical cystectomy and, if chosen, the ileal neobladder were carried out applying the technique published by Hautmann et al. [10]. Contradictions for continent urinary diversion were impaired renal function (serum creatinine >2 mg/dL), severe hepatic dysfunction, and intestinal disease/tumor manifestation as well as tumor invasion in the urethral margin. Ileal conduit (Wallace I) was conducted in the standard fashion using the minimum amount of ileum.

### 2.3. Postoperative Course

Patients were postoperatively managed in the ICU and recovery room. Further postoperative management was carried out on the urological ward according to our standardized clinical care pathways for cystectomy. This involved removal of the gastric tube immediately after surgery, and standard mobilization and standard nutritional buildup. On the first and second postoperative days, patients received an increase of liquid food (up to 500 mL water, up to four cups of yogurt). A gradual buildup with solid food components and full mobilization were initiated on the third postoperative day. All occurrences requiring a surgical intervention or medication were assessed as a complication. Ileus was defined as postoperative nausea or vomiting associated with abdominal distension requiring cessation of oral intake and intravenous fluid support and/or nasogastric tube (NGT) placement by postoperative day 5 resulting in patient fasting with or without NGT placement or antiemetic medication administration.

### 2.4. Questionnaire on Quality of Life

Quality of life was analyzed using the EORTC QLQ-C30 questionnaire [11]. This was supplemented by the QLQ-BLM30 module of the EORTC, which was developed specifically for patients with muscle-invasive bladder cancer. QLQ-BLM30 has completed Phase 3 of the module development and was provided by the EORTC for this investigation (Dr. N. Aaronson, Project Leader, The Netherlands Cancer Institute, Department of Psychosocial Research and Epidemiology, Plesmanlaan 121, 1066 CX Amsterdam, The Netherlands). The questionnaires were analyzed for the QLQ-C30 questions according to the instructions in the EORTC scoring manuals [12]. The EORTC scoring system was also used to assess the QLQ-BLM30 module.

### 2.5. Statistical Data Analysis

SPSS 16.0 software was used for statistical analysis. All interval scale data were assessed by the Kolmogorov-Smirnov test with Liliefors significance correction for deviations from normal distribution. Significance was determined by using either the two-sided *t* test, the two-sided Mann-Whitney *U* test, Pearson's chi-square test, or Fisher's exact test. The Kaplan-Meier procedure with the log-rank test was used for survival statistics. An error probability of *P* < 0.05 was defined as the significance limit.

## 3. Results

### 3.1. Patient Collective and Followup

The median age was 70 years (interquartile range, 64–75) in patients with an ileal conduit (group1) and 62 years (56–66) in group 2 patients (*P* < 0.001). The mean observation time was 33.2 ± 32.77 months in group 1 and 50.6 ± 44.98 months in group 2. Median comorbidity grade in patients with ileal conduit and ileal neobladder was 3 (range 2-3) and 2 (range 2-3), respectively (*U*-test; *P* = 0.041). The pathological tumor stages were evaluated by assignment to three prognostic groups: organ confined (≤pT2, pN0), non-organ confined (≥pT3, pN0), and lymph node positive (pN+) [13, 14]. This distribution differs to a highly significant degree between groups 1 and 2 (2-sided *χ* ^2^ test, *P* < 0.001). No patient in either group received neoadjuvant chemotherapy or radiation therapy. Adjuvant chemotherapy was administered in 34 (24%) patients in the ileal conduit group and in 29 (20%) in the orthotopic diversion group (*P* = 0.509). Adjuvant radiotherapy was administered in 3 patients of each group (*P* = 0.849). Patients' characteristics and their individual therapeutic approaches are summarized in Table 1.

### 3.2. Early Complications

All in-hospital events were assessed as early complications (Table 2). The most frequent complications in group 1 were wound related (20.7%), infectious (13.1%), and pulmonary (13.1%), while gastrointestinal (16.5%), wound-related (15.7%), and infectious (12.2%) complications were most common in group 2. The two groups differed significantly with regard to the occurrence of postoperative ileus (*P* = 0.0184. Fisher's exact test) but not with regard to any of the other complications measured (Table 2). Perioperative mortality (within the first 30 days after surgery) was 4.1% in group 1 and 1.7% in group 2 (*P* = 0.47, Fisher's exact test). The reoperation rate was 17.1% in group 1 and 10.4% in group 2 (*χ* ^2^ test, *P* = 0.124).

### 3.3. Quality of Life

In 2008 we sent the quality of life questionnaires to 126 surviving patients, of whom 58 completed and returned the survey for a 46% response rate. Data obtained with the QLQ-C30 questionnaire disclosed three significant differences between patients of group 1 and 2 (Table 3). The global health status/quality of life (72.3 ± 19.5, *n* = 34 versus 58.0 ± 25.3; *n* = 23; *P* = 0.019, 2-sided *t*-test;) and physical functioning (82.6 ± 19.9, *n* = 34 versus 65.8 ± 29.4, *n* = 24; *P* = 0.018, 2-sided *U* test) were rated markedly higher by patients with ileal neobladder (group 2). All other functions (role functioning, emotional, cognitive, and social functioning) are also better performed by patients with ileal neobladder than by those with ileal conduit but did not reach statistical significance. With one exception, all symptom scales show higher values for patients with ileal conduit without statistical significance (group 1). Diarrhoea is the only symptom that occurs significantly more often in patients with ileal neobladder than in those with ileal conduit (25.5 ± 31.3, *n* = 24 versus 4.2 ± 14.9, *n* = 34); *P* = 0.004, 2-sided *U* test). The two patient groups did not differ significantly in the results of the QLQ-BLM30 module (Table 4). Sexual functioning could not be analyzed because an altogether insufficient number of patients answered these questions.

### 3.4. Overall Survival and Relapse

In 180 patients (69.0%) sufficient follow-up data were available for Kaplan-Meier analysis. OS differed significantly between the ileal neobladder and the ileal conduit group (log-rank test; *P* < 0.001). The estimated 5-year survival was 46% and 67% in groups 1 and 2, respectively. The estimated 10-year survival was 30% in group 1 and 56% in group 2. Survival correlated significantly with the prognostic group (pathological tumor stage) in the total patient population (*P* < 0.001, log-rank test). For the entire study population the 5-year survival probability was highest in patients with an organ-confined (≤pT2, pN0) tumor (82%) and markedly lower in those with a non-organ-confined (≥pT3, pN0) tumor (48%) or a lymph-node-positive (pN+) tumor (28%), respectively (*P* < 0.001). When 5-year OS was compared according to prognostic groups, patients with ileal neobladder had a significantly higher survival in patients with organ-confined (≤pT2, pN0) tumors (80%) than those with ileal conduit (70%) (log-rank test; *P* = 0.03). In the other two prognostic groups, that is, ≥pT3, pN0 patients and pN+ patients, the 5 year OS rate did not differ significantly between patients with either urinary diversion. The survival probability was not influenced by the different comorbidity grades of the study population (group 1, *P* = 0.879; group 2, *P* = 0.474). The relapse rate was 34% in group 1 and 26% in group 2 (2-sided *χ* ^2^ test; *P* = 0.26, Table 1). Systemic relapses occurred in both groups (group 1: 61.3%; group 2: 40%; 2-sided *χ* ^2^ test; *P* = 0.23). Local relapse occurred in 5 patients in group 1 and in 7 patients in group 2 (2-sided *χ* ^2^ test; *P* = 0.23).

## 4. Discussion

As the risk for developing any type of complications in both sexes ranges from 16% to 66%, radical cystectomy with neobladder reconstruction represents a major, complication-prone surgery [15–17]. This wide range of complication rates reported by different studies is mainly explained by the fact that various complications are not surveyed in detail in different standardized reporting systems, and studies are thus difficult to compare. In our series, ileus occurred significantly more often in patients with ileal neobladder. In contrast to our data, Parekh et al. [5] found a higher incidence of postoperative paralytic ileus in patients with ileal conduit (7.4%) than in those with neobladder (2.6%). Nieuwenhuijzen et al. reported a very similar incidence of postoperative ileus for both urinary diversions [15]. The heterogeneous results of the published studies suggest that the reason for postoperative ileus is manifold and can occur regardless of the type of urinary diversion [8]. However, one explanation for the higher incidence of colonic motility disorders in the ileal neobladder group might be leakage of urine from the not-yet fully healed neobladder in the early postoperative phase [16].

Many different questionnaires are used to measure the quality of life in oncological urology (e.g., self-developed questionnaires, SF-36, FACT-G, and QLQ-C30). Thus it is very difficult to achieve comparability of results [18]. Not all the questionnaires have been validated, and they differ considerably in some of the topics covered. Another difficulty lies in the fact that quality of life is a multidimensional concept incorporating different domains that are weighted by their importance to the individual and may change over time [19]. In oncological urology, quality of life is usually assessed in retrospective studies where only postoperative data are available in most cases [20–22]. An interaction could even be demonstrated between patients and the investigating institution, which is hypothetically attributed to heroization and idealization of the attending urologists [23].

We used the validated EORTC QLQ-C30 questionnaire with the QLQ-BLM30 module for the present investigation in order to exclude as many of the above-mentioned problems as possible. Analysis of the QLQ-C30 questionnaire showed that patients with ileal conduit (group 1) differed significantly from those with ileal neobladder (group 2) in the assessment of the general health status and quality of life and in the mastery of physical functions. These findings were in line with the results obtained by Hobisch et al. using the same questionnaire [6]. In our series the occurrence of diarrhoea differed significantly between patients of group 1 and 2 favouring patients with an ileal conduit. This observation was not experienced by other studies with the QLQ-C30 questionnaire that show no significant differences between the two surgical procedures [24, 25].

In our study, the assessment of the QLQ-BLM30 module showed no significant differences between patients of group 1 and 2, which also coincides with the results of another analysis [24]. Nevertheless, the application of the SF-36 questionnaire in other studies comparing patients with orthotopic diversion versus ileal conduit yielded a heterogeneous picture in terms of the subjective quality of life assessment [26–29].

The 5-and 10-year overall survival was analyzed to be significantly higher in patients who received an ileal neobladder compared to those with an ileum conduit (*P* < 0.001). However, this observation has to be interpreted with great caution since both groups are heterogeneous in age, comorbidity, and especially in distribution to well-known prognostic parameters such as tumor stage and lymph node status (*P* < 0.001, 2-sided *χ* ^2^ test). This could, of course, also account for the differences in postoperative quality of life. The heterogeneity of the study groups clearly demonstrates that although the patient has great influence on the definite type of diversion, the surgeons' preference and information remain the important determinant of choice, considering the differences in age and comorbidity between the patients opting for an ileal conduit and continent diversions. Other limitations of our study are certainly its retrospective design and heterogeneous followup, partly with a long follow-up period. Obviously perioperative patient care and methods of patient counseling have been steadily improved and changed over this 14-year period and might have affected our results. Due to the nonrandomized retrospective nature of our study based on two heterogeneous cohorts, prospective randomized trails would be needed to prove real value. However, we are aware that it is extremely unlikely that a prospective randomized trial comparing ileal conduit to neobladder can and will ever be realized.

## 5. Conclusions

Cystectomy with any type of diversion remains a complication-prone surgery. Even if the patient groups are not homogeneous in most respects, there are many arguments in favor of the ileal neobladder as the urinary diversion of choice whenever technically feasible and oncologically justified.

## Figures and Tables

**Table 1:** Demographic and pathological characteristics.

Characteristics	Ileal conduit Median (interquartile range) abs. incidence/n, rel. (%)	Ileal neobladder Median (interquartile range) abs. incidence/n, rel. (%)
Number	146	115
Age	70 (64–75)	62 (56–66)
Gender		
male	98/146 (67%)	110/115 (96%)
female	48/146 (33%)	5/115 (4%)
Comorbidity grade^a^	3 (2-3)	2 (2-3)
Pathological tumor stage^b^		
organ confined ≤pT2, pN0	46/145 (32%)	65/115 (57%)
non-organ confined ≥pT3, pN0	51/145 (35%)	30/115 (26%)
Lymph node- positive pN+	48/145 (33%)	20/115 (17%)
Adjuvant therapy		
Chemotherapy	34/146 (24%)	29/115 (20%)
Radiation therapy	3/146 (2%)	3/115 (3%)
Data on relapse (number of patients)	100	80
Relapse	34/100 (34%)	21/80 (26%)

^a^ *U* test; *P* = 0.041.

^b^2-sided *χ* ^2^ test, *P* < 0.001.

**Table 2:** Early complications.

Early-onset complications	Ileal conduit abs. incidence/n rel. incidence (%)	Ileal neobladder abs. incidence/n rel. incidence (%)
Number of patients	146	115
Gastrointestinal	10/145 (6.9%)	19/115 (16.5%)
ileus^a^	8/145 (5.5%)	17/115 (14.8%)
rectal injury	2/145 (1.4%)	2/115 (1.7%)
Infectious	19/145 (13.1%)	14/115 (12.2%)
sepsis	13/145 (9.0%)	9/115 (7.8%)
intraabdominal/pelvic abscess	6/145 (4.1%)	5/115 (4.3%)
Wound	30/.146 (20.7%)	18/115 (15.7%)
Infection/dehiscence	30/.146 (20.7%)	18/115 (15.7%)
Urinary tract leakage (urinoma)	3/145 (2.1%)	8/115 (7.0%)
Pulmonary	19/145 (13.1%)	11/115 (9.6%)
pneumonia	19/145 (13.1%)	11/115 (9.6%)
Thromboembolic	10/145 (6.9%)	2/115 (1.7%)
thrombosis	6/145 (4.1%)	1/115 (0.9%)
pulmonary artery embolism	4/145 (2.8%)	1/115 (0.9%)
Lymphocele	10/145 (6.9%)	8/115 (7.0%)
Reoperations	25/146 (17.1%)	12/115 (10.4%)
Overall complication rate^b^	69.1%	69.6%

^a^Fisher's exact test *P* = 0.018.

^b^Percentage of patients with one or more complications.

**Table 3:** QLQ-C30.

QLQ-C30	Ileal conduit Mean ± standard dev. Median (interquartile range) *n* = number of cases	Ileal neobladder Mean ± standard dev. Median (interquartile range) *n* = number of cases
*Functional scales*		
Global health status/quality of life^a^	58.0 ± 25.3	72.3 ± 19.5
	58.3 (33.3–83.3)	70.8 (56.2–85.4)
	*n* = 23	*n* = 34
Physical functioning^b^	65.8 ± 29.4	82.6 ± 19.9
	70 (33.3–93.3)	93.3 (71.6–100)
	*n* = 24	*n* = 34
Role functioning	63.8 ± 31.1	76.0 ± 27.9
	66.7 (33.3–100)	83.3 (50–100)
	*n* = 24	*n* = 34
Emotion functioning	72.2 ± 22.3	81.1 ± 22.3
	70.8 (52.1–91.7)	87.5 (66.7–100)
	*n* = 24	*n* = 34
Cognitive functioning	77.8 ± 22.9	83.3 ± 20.5
	83.3 (54.2–100)	83.3 (66.7–100)
	*n* = 24	*n* = 34
Social functioning	65.3 ± 32.2	70.1 ± 33.0
	83.3 (33.3–95.8)	75 (45.8–100)
	*n* = 24	*n* = 34
*Symptoms scales*		
Fatigue	37.5 ± 28.1	26.0 ± 28.3
	33.3 (11.1–63.9)	16.7 (0–55.6)
	*n* = 24	*n* = 34
Nausea and vomiting	9.7 ± 20.2	3.4 ± 12.8
	0 (0–12.5)	0 (0-0)
	*n* = 24	*n* = 34
Pain	26.4 ± 31.8	18.6 ± 34.0
	8.3 (0–50)	0 (0–33.3)
	*n* = 24	*n* = 34
*Single items*		
Dyspnoea	37.5 ± 35.9	27.5 ± 37.1
	33 (0–66.7)	0 (0–41.7)
	*n* = 24	*n* = 34
Insomnia	29.2 ± 31.6	21.6 ± 27.1
	33.3 (0–66.7)	0 (0–33.3)
	*n* = 24	*n* = 34
Appetite loss	18.1 ± 31.1	6.9 ± 17.9
	0 (0–33.3)	0 (0-0)
	*n* = 24	*n* = 34
Constipation	22.2 ± 30.6	11.8 ± 19.9
	0 (0–33.3)	0 (0–33.3)
	*n* = 24	*n* = 34
Diarrhoea^c^	4.2 ± 14.9	23.5 ± 31.3
	0 (0-0)	0 (0–33.3)
	*n* = 24	*n* = 34
Financial difficulties	25.0 ± 35.8	20.6 ± 32.8
	0 (0–66.7)	0 (0–41.7)
	*n* = 24	*n* = 34

^a^ *t*-test; *P* = 0.019.

^b^ *U* test; *P* = 0.018.

^c^ *U* test; *P* = 0.004.

EORTC QLQ-C30 results of this study. The questionnaire assesses cancer-specific QOL. For all the questions, a scale from 1 to 4 was used (1: not at all, 2: a little, 3: quite a bit, 4: very much). All scores were linearly transformed such that all scales range from 0 to 100. For the six functional items, the higher score represents a higher level of functioning and for the symptoms/single items, a higher score means a higher level of symptomatology/problems.

**Table 4:** QLQ-BLM30

QLQ-BLM30	Ileal conduit Mean ± standard dev. Median (interquartile range) *n* = number of cases	Ileal neobladder Mean ± standard dev. Median (interquartile range) *n* = number of cases
Urinary symptom		33.6 ± 26.3
		33.3 (9.5–52.4)
		*n* = 33
Urostomy problem	25.6 ± 22.0	
	19.4 (6.9–43.8)	
	*n* = 24	
Single catheter use problem	0 ± 0	6.7 ± 14.9
	0 (0-0)	0 (0–16.7)
	*n* = 1	*n* = 5
Future perspective	39.1 ± 33.8	24.3 ± 27.9
	33.3 (11.1–66.7)	11.1 (0–44.4)
	*n* = 23	*n* = 32
Abdominal bloating and flatulence	28.3 ± 25.8	29.7 ± 31.0
	33.3 (0–50.0)	16.7 (0–50)
	*n* = 23	*n* = 32
Body image	34.1 ± 32.3	33.5 ± 28.2
	22.2 (11.1–55.6)	33.3 (11.1–55.6)
	*n* = 23	*n* = 32
Sexual functioning	no available data	no available data

EORTC QLQ-BLM30 results of this study. The questionnaire is a phase-3 module that specifically evaluates the impact of radical cystectomy and reconstructive surgery in terms of health-related quality of life. For all the questions, a scale from 1 to 4 was used (1: not at all, 2: a little, 3: quite a bit, 4: very much). All scores were linearly transformed such that all scales range from 0 to 100. For the symptoms/single items, a higher score means a higher level of symptomatology/problems.
